# Crucial Obstacles and Strategies for Human RSV Pediatric Vaccine Development

**DOI:** 10.3390/v18010036

**Published:** 2025-12-24

**Authors:** Chen Ling, Yuya Wang, Rui Xiong, Yong Wu, Susu Liu, Weijia Li, Yining Wang, Yuwei Zhao, Changfa Fan

**Affiliations:** 1 Division of Animal Model Research, Institute for Laboratory Animal Resources, National Institutes for Food and Drug Control (NIFDC), Beijing 102629, China; lingchen9403@163.com (C.L.);; 2 Department of Microbiology & Infectious Disease Center, School of Basic Medical Sciences, Peking University Health Science Center, Beijing 100083, China; 3 College of Life Science, Northwest University, Xi’an 710069, China; 4 Provincial Key Laboratory of Biotechnology of Shaanxi Province, Xi’an 710069, China

**Keywords:** human respiratory syncytial virus (hRSV), pediatric vaccine development, obstacles and strategies, enhanced RSV disease (ERD)

## Abstract

Human respiratory syncytial virus (RSV) remains a leading cause of severe lower respiratory tract infections in infants and immunocompromised populations, causing approximately 160,000 annual deaths globally. Despite recent approvals of prefusion F (pre-F) protein-based vaccines (Arexvy, Abrysvo) for older adults and pregnant women, pediatric vaccine development faces unique challenges including enhanced respiratory disease (ERD) risks, maternal antibody interference, and immature infant immune responses. Meanwhile, G protein glycosylation variability and NS1/NS2-mediated interferon suppression remain the outstanding difficulties in structure-based vaccine design. Additionally, current animal models demonstrate notable constraints in virus replication, host susceptibility, immune responses, clinical symptoms, and ERD phenomena. This review synthesizes current obstacles and innovative strategies, highlighting that the selection of multi-antigen strategies, appropriate adjuvants, and the development of more precise preclinical animal models are critical elements that will determine the efficacy and safety of future RSV vaccines.

## 1. Introduction

Human respiratory syncytial virus (RSV) infection is one of the most significant health concerns in infants, the elderly and people with poor immune function [1,2]. It has also been included among the top 17 endemic pathogens for which new vaccines are urgently needed, according to a new World Health Organization (WHO) study [3,4]. RSV, transmitted through saliva and droplet spread, causes approximately 64 million acute respiratory infections (ARIs) and 160,000 deaths annually, imposing a substantial global burden [5,6,7]. Nearly all children are infected with RSV by the age of two years and then repeatedly infected throughout life [8,9]. Specifically, it is globally reported to be responsible for nearly 100,000 deaths among children less than five years old, accounting for 65% of RSV-attributable deaths in this cohort [10,11]. Up to 40% RSV infected children will develop a Lower Respiratory Tract Infection (LRTI) [12], which has been associated with long-term pulmonary sequelae, such as asthma [13], for up to 30 years after infection [14]. Overall, RSV infections are recognized as a significant contributor to healthcare and economic burdens globally, as they frequently necessitate hospitalization and intensive care, resulting in substantial utilization of health systems [15].

RSV associated bronchiolitis follows well-documented seasonal peaks and spread more efficiently in colder temperatures [16]. RSV infection manifests with a spectrum of clinical presentations, ranging from mild upper respiratory tract symptoms to severe bronchiolitis and pneumonia that require supportive care [17]. The further spread of infection into distal airway regions increases mucus production and inflammation, leading to a narrowing of the airway that results in bronchiolitis in young children and ARIs in older adults or those with underlying chronic conditions [18,19].

RSV pathogenesis is complex, involving initial epithelial cell invasion and viral replication, followed by an exaggerated immune-mediated inflammatory response [20]. Despite decades of research on RSV, many immune mechanisms remain to be explored [21]. In this situation, prioritizing the development of an RSV vaccine is imperative. Recently, two structure-based RSV prefusion F vaccines (Arexvy, GlaxoSmithKline; Abrysvo, Pfizer) developed to target older and pregnant individuals [1,22,23] have been approved, but none are currently available for children [24,25]. The challenges associated with RSV vaccine development encompass several key aspects: concerns remain regarding the potential of non-live virus vaccines to predispose young children to enhanced RSV disease (ERD); the challenge of achieving an optimal balance between sufficient attenuation to ensure safety and adequate viral replication capacity required to induce robust immunogenicity and protective efficacy in live attenuated vaccines (LAVs); the difficulty in inducing and evaluating protective immunity; and the balance between cell-mediated immunity and humoral immunity. According to the WHO guideline, it is essential to distinguish between RSV-naïve and RSV-experienced individuals, and to prioritize safety assessment in the reinfection population during the development and evaluation of pediatric RSV vaccines.

Given that children are the cohort most in need of protection, and that RSV pediatric vaccine developments are hindered, in this review, we provide a concise overview of the challenges in RSV vaccine development and the current strategies to address them. We further provide an in-depth analysis of the unique obstacles in pediatric RSV vaccine development and highlight key considerations for pediatric RSV vaccine evaluation, aiming to inform and support future research and development efforts.

## 2. Valuable Lessons Learned from the RSV Vaccine Development and Research

Inhalation therapy and corticosteroids have been utilized in the management of acute RSV-LRTIs [26,27,28]. Except for supportive care for symptoms mentioned above, the focus has been the prevention of RSV either by way of passive or active immunization [14]. Maternal immunization, active immunization of older infants and toddlers and the administration of long acting mAbs to neonates and infants are the major preventative strategies for the protection of infants against RSV [29]. Different vaccine types under investigation for the prevention of RSV include live attenuated virus vaccines, chimeric vaccines, protein-based vaccines, including nanoparticles, nucleic acid vaccines and recombinant vector-based vaccines (Table 1). This section presents a comprehensive review of the aforementioned vaccines, focusing on the challenges in RSV vaccine development and the corresponding strategies to address them.

### 2.1. The Immune Evasion Challenges Associated with the Stability and Diversity of Viral Proteins

**Figure 1:**
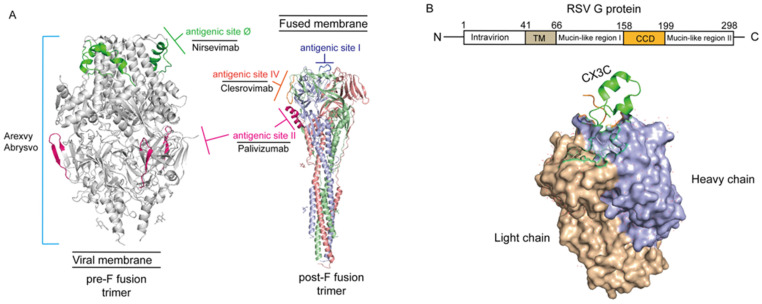
The structure of G and F protein of the RSV virion (PDB code: 7UJA and 3RRR). (**A**) Neutralizing epitopes on RSV pre-F and post-F fusion. The image information is derived from Gilman et al. 2016 and McLellan et al. 2013 [47,48]. Antigenic site II (marked light pink) was solvent exposed in both pre-fusion and post-fusion conformations compatible with antibody binding, and corresponds to the epitope recognized by Palivizmab [48]. Antigenic site Ø (marked green), located exclusively at the apex of the native, intact prefusion F protein trimer, is the most potent neutralizing epitope, and corresponds to the epitope recognized by Nirsevimab. Those epitopes on pre-F fusion form the immunological foundation for all currently approved prefusion-stabilized F protein-based vaccines, including Arexvy and Abrysvo (light blue lines). Otherwise, antigenic site I (marked heavy blue) and IV (marked orange) also are the main epitopes of post-F fusion. Antigenic site IV corresponds to the epitope recognized by Clesrovimab. (**B**) Schematic diagram of RSV G protein structure. The image information is derived from Yu et al. 2016 [49]. The light chain of RSV G protein was marked by brown and heavy chain was marked by violet-blue. The N-terminus of RSV G protein located intracellularly and C-terminus extracellularly, anchored to the viral envelope via the TM domain. The conserved central domain (CCD) spans amino acid residues 158–199 and contains a CX3C motif (marked green). The flanking mucin-like regions are highly variable in sequence and extensively glycosylated.

The adhesion protein (G protein) and fusion protein (F protein) are the major glycoproteins on the surface of the RSV virion, which control the initial phases of infection (Figure 1). The F protein represents the primary target for antiviral drug development, while both the G and F glycoproteins serve as key antigens recognized by neutralizing antibodies elicited during natural infection [47,48,49].

The RSV F protein is the most abundant enveloped protein and is highly conserved across various viral strains [50]. It serves as the primary target for RSV vaccine development due to its critical role in mediating virus cell-to-cell transmission and syncytium formation [51]. The F protein exists in two functional conformations, pre-F and post-F [52]. Specifically, the pre-F conformation contains key neutralizing epitopes (the V and Ø sites); however, it is inherently unstable under native conditions and readily undergoes transition to the post-F conformation, resulting in diminished immunogenicity [53]. Stabilizing the F protein in its pre-F conformation remains a fundamental challenge in the design and development of RSV vaccines [54]. Structure-based rational antigen design, including DS-Cav1, SC-TM, sc9-10 DS-Cav1, and 847A, has yielded significant advances in stabilizing the prefusion F (pre-F) protein, with numerous preclinical and clinical studies demonstrating favorable immunogenicity and safety profiles of pre-F-based vaccine candidates [55].

Three approved RSV vaccines are all pre-F-based. First of all, Arexvy is an adjuvanted subunit vaccine and the world’s first approved RSV vaccine for individuals aged 60 and above [56]. The antigen utilized in Arexvy is the recombinant RSV pre-F protein, which is combined with the AS01E adjuvant [57]. This has enhanced the specificity of RSV-specific CD4^+^ T cells in the elderly, overcome age-related natural immunity decline, and raised the level of neutralizing antibodies in the body, providing protection against RSV infection and related diseases at a rate of 82.6% [40,57]. On 31 May 2023, the FDA approved Pfizer’s Abrysvo vaccine for the elderly to prevent A and B subtypes of RSV [22,43]. Similar to Arexvy, Abrysvo also contains a stable F antigen but differs in being glycosylated [22]. Additionally, the vaccine offers some protection for infants. However, it cannot be used for high-risk pregnant women as it might expose their children to a higher risk of RSV-related diseases [23,58]. Pre-eclampsia was the most prevalent adverse event, characterized by high blood pressure in the pregnant mother, and the inherent risks of fetal distress syndrome secondary to hypoxia in the fetus (NCT04424316) [42]. Due to the comparable incidence of neonatal jaundice in the first months of life between the vaccine (7.2%) and placebo groups (6.2%), and given that the overall benefits of preventing RSV-associated LRTI outweigh the potential adverse effects, Abrysvo has been approved by the FDA for the prevention of RSV in adults aged 60 years and older, as well as in pregnant individuals [43,44]. mRNA-1345, manufactured by Moderna, is the third RSV vaccine based on the pre-F form and formulated with lipid nanoparticles (LNPs) to receive approval from FDA on 2024 [59].

Furthermore, the RSV adhesion protein (G protein), a key mediator of initial viral binding to host cells, exhibits extensive glycosylation with significant strain-dependent variability [60]. This glycosylation serves a dual function: steric shielding of viral epitopes by means of carbohydrate side chains, thereby limiting antibody accessibility, and modulation of antigenic region conformation, which contributes to immune evasion [61]. Notably, the G protein’s high proline content promotes an extended conformation, further contributing to its antigenic plasticity [60]. The G protein contains two hypervariable regions (HVR1 and HVR2), with HVR2 exhibiting significant length polymorphisms (e.g., 72-nucleotide repeats in ON1 or 60-nucleotide repeats in BA subtypes) [62]. These variations drive viral subtyping (A/B) and further classification into subtypes (e.g., NA1-NA4, BA1-BA12) [62]. Additionally, glycosylation masks conserved epitopes or induces conformational changes, further impairing neutralization by antibodies [63]. Despite proposed strategies involving multivalent antigenic chimeras to enhance cross-protection, significant challenges remain. Firstly, the distribution and patterns of glycosylation sites vary across subtypes, complicating the design of chimeric antigens that accurately mimic native structures [64]. Meanwhile, the combination of proline-rich regions and hypervariable domains in the G protein limits the accessibility of broadly neutralizing epitopes [65]. The pace of antigenic drift often outpaces vaccine development efforts, rendering chimeric designs obsolete against emerging strains [66]. Thus, the combined use of anti-G and anti-F antibodies to enhance passive immunity may represent a promising strategy in future vaccine development [67].

Notably, NS1/NS2 can suppress the host interferon signaling pathway and attenuate the innate immune response [68]. It may result in a local inhibition of the immune response induced by the vaccine. Specifically, NS1 directly inhibits type I interferon production by sequestering RIG-I and MDA5, essential pattern recognition receptors in the innate immune system [69,70]. Concurrently, NS2 targets the JAK-STAT pathway through proteasomal degradation of STAT2, effectively neutralizing interferon-mediated antiviral responses [71]. This dual inhibition creates an immunologically permissive microenvironment that facilitates viral replication, particularly in airway epithelial cells where RSV establishes primary infection [72]. The functional consequences of these mechanisms manifest in three primary challenges for vaccine development: first, mucosal immune suppression in vaccinated individuals permits breakthrough infections despite systemic immunization; second, conventional antibody responses targeting surface glycoproteins fail to counteract NS1/NS2-mediated intracellular immune suppression; third, sequence polymorphisms in NS2 across RSV subtypes complicate the development of broadly effective inhibitors [73,74].

Emerging therapeutic strategies demonstrate limited efficacy. For instance, small-molecule inhibitors like ribavirin analogs only partially restore STAT2 stability [75], while CRISPR-based approaches targeting NS1/NS2 transcripts face substantial delivery challenges. Consequently, the development of next-generation RSV vaccines requires a comprehensive and integrated immunological approach to effectively address viral immune evasion, ensure durable protection, and mitigate the risk of vaccine-enhanced disease [41]. One promising approach is the development of universal vaccines that target conserved epitopes within the fusion domain of the F protein to address strain variability [64]. Another direction involves multi-pathogen combination vaccines, such as rBCG-N-hRSV or RSV and human metapneumovirus (hMPV) recombinant vaccine, which could simplify immunization against co-circulating respiratory pathogens [76,77]. A key challenge for both strategies lies in minimizing antigenic interference while preserving the immunogenicity, efficacy and safety of each component [64]. Future designs should therefore consider incorporating specific cytotoxic T lymphocyte epitopes targeting NS1/NS2 to enable the pre-emptive clearance of infected cells [78]. Additionally, the use of Toll-like receptor agonist adjuvants may help compensate for impaired interferon signaling by activating alternative immune pathways.

### 2.2. ERD Risk Assessment

The setbacks in early RSV vaccine development primarily stemmed from insufficient understanding of immune pathological mechanisms [79]. Clinical trials of the formaldehyde-inactivated RSV vaccine (FI-RSV) in the 1960s revealed that vaccinated children exhibited abnormally severe pulmonary inflammation upon natural infection [80], characterized by eosinophil infiltration in lung tissue and peribronchial lymphocyte aggregation [81]. Subsequent animal experiments confirmed that while FI-RSV-induced antibodies could neutralize the virus, they lacked protective IgG subclasses and failed to activate mucosal immunity, leading to complement-mediated immune complex deposition in lung tissue [82]. This immune deviation phenomenon resulted from conformational changes in vaccine antigens, which disrupted neutralizing epitopes while T-cell epitopes of the F protein still stimulated CD4^+^ cells to produce Th2 cytokines such as IL-4/IL-10 [83], ultimately triggering delayed-type hypersensitivity [84,85,86,87,88]. This case highlighted the potential risks of traditional inactivated vaccines for respiratory viruses like RSV: relying solely on humoral immune responses might disrupt the Th1/Th2 balance and exacerbate disease progression [89,90]. Moreover, the phenomenon in which cytotoxic T lymphocyte (CTL) epitopes that are essential for viral clearance are masked, whereas B cell epitopes promoting non-neutralizing antibodies dominate the immune response, underscores the necessity of antigen design targeting both the humoral and cellular immune pathways concurrently [91].

At present, RSV vaccine development has achieved dual optimization of immunogenicity and safety through synergistic design of antigen conformation stabilization, balanced epitope selection, and novel adjuvant systems [79]. At the antigen design level, stabilizing the pre-F protein conformation has emerged as a critical breakthrough, with engineered CTL epitopes now strategically integrated alongside B-cell epitopes to restore immune balance [10]. For example, Arexvy employs the AS01B adjuvant system, whose core components-MPL (monophosphoryl lipid A) and QS-21 saponin-specifically activate the TLR4 pathway and promote dendritic cell maturation, thereby inducing Th1-type immune responses [56]. Clinical data show that the AS01B adjuvant can increase pre-F-specific antibody titers by more than 10-fold while promoting IFN-γ-secreting CD4^+^ and CD8^+^ T cell proliferation [56,92]. This Th1/CTL synergistic response pattern closely resembles the protective immunity generated by natural infection, where both neutralizing antibodies and viral clearance mechanisms operate in concert [93]. Additionally, next-generation adjuvants like Matrix-M^TM^ further enhance mucosal IgA responses by mimicking virus-like particle structures, addressing the deficiency of early vaccines in respiratory local immunity [94]. This optimized antigen-adjuvant synergy strategy not only resolves the immune pathology issues of traditional vaccines but also provides new insights for combating RSV antigen diversity through carefully balanced cellular and humoral immune activation.

### 2.3. Horizontal Comparison of Technical Routes for Pediatric RSV Vaccination: Balancing Immunogenicity and Safety

The immunological landscape of early childhood is characterized by an immature immune system, the presence of maternally derived antibodies (MDAs), and heightened susceptibility to severe respiratory infections [95]. Consequently, a rigorous assessment of the balance between immunogenicity and safety is essential in pediatric vaccine development.

mRNA vaccines represent a transformative platform validated during the COVID-19 pandemic [96]. In adults, mRNA-1345 demonstrated 83.7% efficacy against RSV-associated LRTI, driven by the robust induction of neutralizing antibodies and Th1-biased cellular immunity (Table 1) [36]. The advantages of mRNA vaccines encompass the rapid induction of antibody production, effective targeting of key neutralizing epitopes on the pre-F protein to elicit specific immune responses, and circumvention of the risk of RSV vaccine-associated ERD [97]. However, due to the intrinsic reactogenicity of LNPs employed in delivery systems, they frequently trigger transient local and systemic adverse events—such as fever, a particularly sensitive safety endpoint in the infant population [98]. mRNA instability, ultra-low storage requirements, and LNP liver accumulation are also key persisting hurdles faced in mRNA vaccine development [99]. Moreover, the application of mRNA vaccines in children remains confronted with substantial challenges, necessitating rigorous age de-escalation studies to systematically characterize their reactogenicity profiles.

RSV LAVs represent an immunization approach that closely mimics natural infection, eliciting a balanced and protective immune response by simulating pathogen invasion while avoiding significant clinical disease [37]. The primary advantage of the LAVs lies in its ability to elicit a robust systemic immune response while simultaneously inducing mucosal immunity at key sites of pathogen entry, such as the respiratory tract [38]. This includes the production of secretory IgA (sIgA), which serves as a critical first line of defense against viral invasion [100]. Furthermore, LAVs are generally capable of overcoming interference from maternal antibodies, enabling the establishment of active immunity during early infancy [38]. Historically, LAVs for diseases such as measles and varicella have demonstrated durable and long-term protective immunity in children [24]. However, the development of LAVs faces a central challenge—the precise balance between sufficient attenuation to ensure safety and adequate immunogenicity to confer protection [24,97]. Over-attenuation may result in poor vaccine take and suboptimal immune responses, whereas under-attenuation carries the risk of causing symptomatic upper respiratory illness, rhinorrhea, or residual viral replication in the lower respiratory tract [97]. Current strategies may focus on achieving an optimal attenuated phenotype through approaches such as codon deoptimization or targeted gene deletion; however, these methods are technically demanding and require rigorous assurance of genetic stability to prevent reversion to virulence.

In summary, each vaccine technology platform possesses distinct advantages and concomitant risks. No single platform offers a perfect solution for the pediatric RSV vaccine landscape. Feasible future strategies may encompass the implementation of heterologous sequential immunization regimens, the feasibility of which has been validated in the context of COVID-19 [101,102]. Specifically, this approach could involve administering viral vector vaccines as the primary immunization to overcome the interference of maternal antibodies, followed by booster immunization with subunit vaccines; or individualized selection of vaccine platforms for different subgroups of children based on age, maternal antibody levels, and underlying health conditions. A deep understanding of the unique advantages and limitations of each platform in terms of immunogenicity, safety, and target population is the key foundation for the successful development and scientific deployment of the next generation of RSV vaccines, which is of great significance for effectively protecting infants and young children.

## 3. The Crucial Obstacles and Strategies for Pediatric RSV Vaccine Development

The development of RSV vaccines for infants faces a critical challenge in achieving efficient synergy between mucosal and systemic immune responses [95]. Under physiological conditions, the immune barrier of infant respiratory mucosa requires the simultaneous activation of sIgA and serum IgG responses—the former forms the first line of defense by preventing viral adhesion, while the latter provides systemic protection through neutralizing viral particles and antibody-dependent cellular cytotoxicity (ADCC) [103]. However, existing vaccine technologies exhibit significant limitations: live-attenuated vaccines, though capable of mimicking natural infection and stimulating mucosal-associated lymphoid tissue (MALT) to produce sIgA through viral replication, carry risks of reversion to virulence, restricting their use in immunocompromised populations [104]. In contrast, subunit vaccines based on recombinant proteins, while safer, fail to effectively activate mucosal immunity due to the absence of virus-like particle structures [105]. This immune response imbalance has been validated in animal models—FI-RSV induced high levels of serum IgG but showed significantly fewer sIgA-secreting cells in lung tissue compared to natural infection groups, alongside deficient Th17 cell-mediated mucosal memory responses [106]. Recent studies employing nanoparticle carrier technology (e.g., mRNA-LNP) to target antigen delivery to respiratory mucosa achieved an sIgA/IgG ratio close to natural infection levels in mouse models, though clinical translation still requires the optimization of delivery efficiency and stability [107].

Maternal antibody interference represents another major obstacle to infant vaccine responses [29]. During pregnancy, maternal IgG antibodies transferred to the fetus via the placenta provide passive immune protection in neonates but simultaneously bind to vaccine antigens, forming immune complexes that are cleared, thereby significantly reducing vaccine-induced active immune responses [14]. Clinical data indicate that infants under six months of age vaccinated against RSV exhibit 3- to 5-fold lower neutralizing antibody titers and approximately 40% shorter protection duration compared to unexposed infants [108]. Current solutions primarily involve two strategies: (1) indirect protection through maternal immunization, such as Pfizer’s Abrysvo vaccine, which reduces hospitalization risk by 82% in infants under 6 months when administered in late pregnancy—though this approach fails to establish independent immune memory in infants [22,109]; and (2) antigenic epitope modification technology, such as GSK’s Arexvy, which reduces maternal antibody recognition efficiency by 70% through altered conformation of key neutralizing epitopes, successfully inducing infant immune responses independent of maternal antibodies in rhesus macaque models [56,110]. Notably, a study reported chimeric antigen design fusing RSV F protein with tetanus toxoid, leveraging the latter’s non-competitive binding with maternal antibodies to achieve 89% vaccine efficacy in infants with maternal antibodies [111]. However, these methods remain limited—maternal immunization is constrained by timing windows [112,113], while antigen modifications may impact vaccine immunogenicity [114]. Future research may need to integrate single-cell sequencing to precisely identify binding sites between maternal antibodies and vaccine antigens, providing a molecular basis for rationally designing next-generation vaccines that circumvent interference.

## 4. Absence of Preclinical Animal Models for Predicting ERD

The unpredictable Th2-type immune deviation remains a critical challenge in RSV vaccine development [85]. While modern adjuvants such as AS01E have improved Th1/Th2 modulation, the inherent immunosenescence in elderly populations and immunological naivety in infants continue to pose risks of Th2-biased immune responses [115]. A fundamental limitation lies in the absence of reliable preclinical prediction models capable of forecasting ERD potential [116]. Commonly used animal models in medical research are different types of rodents, as well as different monkey species such as macaques, marmosets and chimpanzees (Table 2). Meanwhile, murine models are widely used in RSV infections, vaccine evaluation assays, due to the cost-effectiveness, accessibility, and availability of a wide range of genetical modified mice and immunological reagents for characterizing immunopathological pathways [117].

### 4.1. Non-Human Primates Models

Chimpanzees have played a crucial role in the discovery and research of RSV and have been used to evaluate the virulence and protective efficacy of live, attenuated RSV vaccine candidates [118]. The antibodies produced by chimpanzees after their first infection with RSV mutant viruses induced protection against subsequent wild-type RSV challenge, while the replication and genetic stability of the mutant viruses parallel that in sero-negative children [119]. Chimpanzees are the only animal model that allowed full permissive to RSV replication, but low levels of neutralizing antibodies and poorly protected against RSV challenge after vaccination of recombinant vaccinia viruses expressing the human RSV F and G, which is contrast with the almost complete protection induced in mice, cotton rats and owl monkeys [120].

Rhesus macaques, African green monkeys and owl monkeys were shown to be susceptible for RSV and exhibited semi-permissive capabilities for RSV replication [121,122]. But those non-human primates have significant limitations in the evaluation of RSV vaccines, mainly due to the lack of clinical and pathology symptoms of RSV infection and the production of lower neutralizing antibody levels in vaccine evaluation [121,122] (Table 2).

### 4.2. Cotton Rat Models

The cotton rat, one of the oldest models for RSV, has been shown to be susceptible to RSV at all ages, from neonatal to adult, exhibiting human-like symptoms and allowing the virus to replicate in both the upper and lower respiratory tracts [123] (Table 2). The virus primarily infects the epithelium, as evidenced by the detection of viral antigens in the nasal, bronchial, and bronchiolar epithelia. The histopathological changes manifest as mild, proliferative bronchiolitis and rhinitis in the affected epithelial tissues. Meanwhile, RSV infection occurred in CX3CR1 positive cells of cotton rats [124]. The localization of infection in cotton rats resembles that of severe disease in humans [125].

Due to the resemblance with human disease, easy to house and spontaneously breed, cotton rats can be used for mother-to-infant transmission studies [126]. For instance, the pregnant female cotton rats vaccinated with LNPs encapsulating the LC2DM mRNA (a modified pre-F protein vaccine, termed LC2DM) not only presented complete protection against RSV infection, without signs of ERD, but also the offspring exhibited robust protection against RSV without presenting with exacerbated lung disease [127]. The application of the pregnant cotton rats has greatly promoted the developments of RSV pediatric vaccines.

Moreover, immunosuppressed cotton rats represented a stronger, more persistent infection as in immunocompromised patients, suggested that cotton rats can serve as a model for all high-risk groups for RSV [128]. However, the short infection cycle, lower viral titers, and milder pathological symptoms in RSV-infected cotton rats limit their utility in simulating the pathogenesis of human RSV infection [129]. The outbred nature of cotton mice present significant challenges for consistency and reproducibility in RSV studies.

### 4.3. Murine Models

The histopathological lung changes in RSV infected BALB/c mice that closely resemble those observed in infected infants, such as interstitial thickening, mononuclear cell infiltration and swelling of the bronchial epithelia [120,130]. BALB/c mice with intermediate susceptibility, have been proven to be a valuable model when investigating vaccine candidates [131] (Table 2). The protective effects of mAb was first demonstrated in BALB/c mice and promoted the development of humanized mAb targeting the RSV F protein [132], ultimately leading to the advent of the Palivizumab prophylaxis. Additionally, BALB/c mice were also used to evaluate the immunogenicity and protective efficiency of the VLP vaccine co-expressing RSV pre-F and Gt antigens [133] and the cold-adapted influenza-vectored RSV vaccine (rFlu/RSV/NA-3F) [134]. This provides a useful tool for vaccine evaluation and proof-of concept.

However, BALB/c mice represent an age-dependent RSV-responsiveness after infection [135]. Older BALB/c mice were shown to be more susceptible to infection, as evidenced by a slight elevation in viral titers and more severe lung lesions and bronchiolitis compared to younger mice [120,136]. Meanwhile, viral antigens were only detected in the alveolar cells, and the presence of RSV antigens in the bronchiolar epithelium was dependent on the inoculation dose [120]. In addition, upper but not lower respiratory tract infection is induced, limiting the assessment of the impact of vaccines on disease severity [137]. Collectively, BALB/c mice are useful as proof-of-concept but with some limitations.

The Rag2^−/−^ mice model with C57BL/6 background well solved the susceptibility problem and supported efficient viral replication of RSV in respiratory organs, including the nasal cavity, trachea, and lungs, which is consistent with both upper and lower respiratory tract infection of clinical patients [129,138]. Moreover, a humanized mouse model with functional human CD4^+^ T cell and B cell (human immune system mouse, HIS mouse) showed weight loss, peribronchiolar inflammation, a predominance of neutrophils in bronchoalveolar lavage fluid (BAL), and enhanced mucus production after 10^6^ pfu of line 19 RSV infection [139]. The phenomenon of up to ~10% neutrophils in BAL was the same as in RSV-infected children [140]. These findings suggest that a humanized mouse model may be a more relevant model for evaluating pediatric RSV vaccine candidates [120], if the problems of high cost and complex model establishment can be properly solved. With the discovery of the RSV functional receptor [141], such types of models should emerge in the future.

So far, a murine model with competent immune response, high susceptibility, and with typical pathological features is urgently needed for vaccine evaluation and is also a blockbuster model tool to promote the progress of RSV pediatric vaccine development [120]. While 7- to 14-day-old mice have been considered as an ideal model to effectively simulate the conditions of infants and young children [142], several factors present significant challenges in constructing and applying RSV pup models, including the limited replication capacity, small size, low tolerance, and specific requirements for infection routes [143]. Moreover, the immature alveolar development in young mice may cause deviations in pulmonary pathological features (such as bronchiolitis and mucus secretion) from human clinical phenotypes [143,144]. Therefore, the optimization of infection routes (such as precise control of intranasal inoculation doses) and the improvement of survival rates during long-term observation are critical [145,146]. Additionally, genetic engineering approaches (e.g., expressing humanized CX3CR1 receptors) and virus adaptive passage to enhance infection efficiency must be considered [147,148].

For instance, while rodent models can assess basic immunogenicity, they lack the immunological complexity to evaluate glycosylation-dependent antigenic changes or predict conformational stability under storage conditions. Non-human primate models, though closer to human responses, are costly and limited in throughput, hindering systematic evaluation of batch variability. This reliance on empirical testing not only depends on development but also heightens the risks of failure, as exemplified by the historical case of the FI-RSV vaccine. Consequently, there is urgent need for a novel rodent model capable of faithfully recapitulating RSV pathology and predicting the risk of ERD, particularly by sensitively detecting vaccine-induced Th2 immune bias. Advanced in vitro systems, such as humanized mice or organoid models, offer promise but require validation for translational relevance. Bridging this gap demands interdisciplinary approaches, integrating structural biology, immunology, and bioinformatics to develop predictive algorithms that correlate molecular stability with clinical outcomes, ultimately ensuring safer and more efficient RSV vaccine development.

## 5. The Inherent Limitations of Production Technologies

The development of RSV vaccines targeting the pre-F protein faces significant challenges in maintaining protein stability and ensuring consistent antigenic properties [52]. The pre-F conformation, which is critical for inducing neutralizing antibodies, is inherently unstable due to its metastable nature. Although mutations such as DS-Cav1 introduce disulfide bonds to stabilize the pre-F structure temporarily [55], large-scale production often leads to conformational conversion to the post-F state, resulting in batch-to-batch variability. This instability poses a major hurdle for vaccine consistency, as even minor structural changes can significantly alter immunogenicity. Additionally, the choice of expression system introduces further complexities. Insect cell-based systems, while cost-effective and scalable, produce F proteins with glycosylation patterns that differ from those in human cells, potentially affecting antigen recognition and immune responses [149]. In contrast, mammalian cell systems, which yield human-like glycosylation, are limited by their high production costs and low yields [51]. These technical challenges highlight the need for advanced stabilization strategies and expression platforms to ensure the reliable production of pre-F-based RSV vaccines. Addressing these issues is essential for developing vaccines that can consistently elicit robust immune responses while maintaining structural integrity throughout manufacturing and storage processes.

## 6. Key Considerations for Future

Efforts to develop RSV pediatric vaccines or immunoprophylaxis remain highly active. The viral immune evasion mechanisms, safety concerns related to ERD, and persistent technical challenges in antigen design and delivery represent enduring obstacles in the development of pediatric RSV vaccines. Moreover, the immunological immaturity of infants and young children also poses significant challenges to the assessment of protective efficacy and conducting clinical trials.

Specifically, the difficulties in assessment of protective efficacy in RSV vaccine development are primarily due to the lack of standardized evaluation criteria across different populations [51]. Current clinical trials predominantly focus on preventing LRTD as the primary endpoint, yet the immunological thresholds required for protection vary significantly between vulnerable groups such as elderly adults and infants [150]. This disparity was starkly illustrated in the Phase III trial of Bavarian Nordic’s MVA-BN RSV vaccine, where the failure to achieve consistent efficacy across age cohorts underscored the need for population-specific benchmarks [151,152]. The absence of consensus on protective correlates of immunity complicates the interpretation of trial outcomes, particularly when vaccines demonstrate variable performance in subgroups [151]. For instance, while some candidates may effectively reduce symptomatic infection in healthy adults, their efficacy in high-risk populations often falls short of regulatory expectations, highlighting the necessity for adaptive clinical endpoint designs that account for age-related immunological differences [150]. This variability not only prolongs development timelines but also increases the risk of late-stage failures, as seen in cases where promising Phase II results failed to translate into broad protection in larger trials (GSK: NCT04605159, NCT04980391, NCT05229068) [153]. Addressing these challenges requires a paradigm shift toward stratified efficacy standards, incorporating biomarkers and immune correlates tailored to distinct demographic categories, thereby ensuring more accurate and meaningful clinical evaluations.

The observed variability in vaccine efficacy extends beyond population-specific challenges, as exemplified by the fluctuating performance of Moderna’s mRNA-1345 vaccine, which demonstrated an initial efficacy rate of 83.7% that later declined to 50% [59,154]. This decline underscores the dynamic nature of immune responses to RSV vaccines, particularly in the context of emerging viral variants or waning immunity over time. Compounding this issue are reports of ERD, where certain formulations paradoxically increased infection severity in preclinical models, mirroring historical concerns with the FI-RSV vaccine [154]. These findings highlight the delicate balance between inducing protective immunity and avoiding aberrant immune responses, necessitating rigorous preclinical screening to identify candidates with optimal safety profiles. Regulatory agencies have responded with heightened caution, as evidenced by the FDA’s temporary suspension of infant vaccine trials pending reevaluation of clinical endpoints [155]. Such interventions reflect growing recognition that traditional efficacy metrics may inadequately capture complex immunological outcomes, particularly in vulnerable populations. In the future, the field must integrate multi-dimensional assessments, including mucosal immunity and cellular responses, alongside conventional serological measures, to better predict real-world vaccine performance. This holistic approach is critical for mitigating risks associated with efficacy fluctuations and ensuring that next-generation RSV vaccines meet both the safety and efficacy standards across diverse populations.

## 7. Conclusions

In conclusion, the development of effective pediatric respiratory syncytial virus (RSV) vaccines represents a formidable scientific challenge that demands interdisciplinary collaboration to overcome the complex interplay between viral biology and host immunity. Maternal antibody interference, the lake of predictive animal models the metastable nature of the pre-F protein, and the antigenic variability of the G protein remain critical challenges in the development of pediatric RSV vaccines. Future success will require concerted efforts in four domains: (1) computational prediction of glycosylation-dependent antigenic drift to guide multivalent vaccine design; (2) development of human-relevant organoid models for ERD risk assessment, such as humanized airway organoids derived from pediatric primary nasal epithelial cells cultured at the air–liquid interface, which recapitulate the human respiratory tract microenvironment and enable the assessment of vaccine-induced immune responses for predicting the risk of ERD; (3) integration of TLR agonist adjuvants to counteract NS1/NS2-mediated interferon suppression; and (4) establishment of correlates of protection tailored to distinct age groups, which should involve the definition of target thresholds for immunobridging markers, including neutralizing antibody titers, mucosal IgA, and antigen-specific T-cell frequencies that are correlated with clinical efficacy in different pediatric age cohorts. Only through such multidimensional innovation can the field achieve the WHO-mandated goal of effective pediatric RSV immunization.

The path forward must balance scientific ambition with rigorous safety oversight. As evidenced by the collaborative efforts behind recently approved vaccines, sustained investment in basic virology, translational immunology, and equitable vaccine access will be paramount to protect vulnerable populations worldwide.

## Figures and Tables

**Table 1:** An overview of the current research on RSV vaccines for pediatric populations.

Antibody and Vaccine	Vaccinated Population	Clinical Trials	Effectiveness	Disadvantages	Refs
**Antibody**					
Nirsevimab	<12 months	Approved by FDA, European Medicines Agency (EMA) and National Medical Products Administration of CHINA (NMPA)	Against hospitalization for RSV-associated bronchiolitis was 83.0% (95% CI, 73.4 to 89.2); against RSV-associated bronchiolitis resulting in critical care was 69.6% (95% CI, 42.9 to 83.8); against RSV-associated bronchiolitis resulting in ventilatory support was 67.2% (95% CI, 38.6 to 82.5).	Pyrexia, discomfort, and local injection-site pain or swelling.	Assid et al. [30] NCT03979313
Palivizumab	Infants and young children born preterm (at or before 35 weeks’ gestation)	Approved by FDA	Preventing confirmed clinical infection was 70% (95% CI, 19–90%); against hospitalization was 82% (95% CI, 29–96%).	Anaphylaxis and some cases of severe hypersensitivity reactions.	Caserta et al. [31] Viguria et al. [32]
Clesrovimab	Infants	Approved by FDA	Against RSV-related hospitalization rates was 84.2% (95% CI: 66.6–92.6, *p* < 0.001); against RSV-related LRIs hospitalization rates was 90.9% (95% CI: 76.2–96.5); against the incidence of severe medically attended LRTIs was 91.7% (95% CI: 62.9–98.1).	The most common adverse event through day 14 was irritability.	Sevendal et al. [33] Zar et al. [34] Syed et al. [35] NCT03524118 NCT04767373
**mRNA vaccines**					
mRNA-1345	≥60 years old 5–8 months	Phase II–III	Against RSV-associated lower respiratory tract disease with at least two signs or symptoms was 83.7% (95.88% CI, 66.0 to 92.2); against the disease with at least three signs or symptoms was 82.4% (96.36% CI, 34.8 to 95.3); against RSV-associated acute LRIs was 68.4% (95% CI, 50.9 to 79.7).	2.8% serious adverse events; half vaccine immunized infants (5 to 8 months) were developed LRTI, with 3 cases being severe or very severe, which was higher than control.	Wilson. et al. [36]
**Attenuated live vaccines**					
RSVt (LID/ΔM2-2/1030s; SP0125)	6–24 months	Phase III	Vaccine efficiency was 85% (90% CI, 78–99.7%); against RSV-medically attended acute respiratory illness was 45% (90% CI, 26–65%).	-	McFarland. et al. [37]
BLB-201	Pediatric (under 2 years of age) Older adult	Phase I	Safe, well-tolerated and no major safety concerns. RSV antibody responses and low replication rate increased in 64% vaccine recipients. RSV neutralizing antibodies increased in 80% vaccine recipients at 4 weeks post-vaccination.	-	Topalidou. et al. [38] NCT05281263
Codavax-RSV	Pediatric	Phase I completed	Safety, well-tolerated. Completed and reached the primary endpoint regarding the safety profile and induced cellular immunity.	-	Topalidou. et al. [38] NCT04295070
MV-012-968	Pediatric	Phase II	Well-tolerated, acceptable safety profile; mild solicited AEs; no SAEs. Neutralizing antibody titers increased rate was 89%.	-	Mazure. et al. [39] NCT04690335
**Subunit vaccines**					
Arexvy	≥60 years old	Approved by FDA	Against the incidence of RSV-LRTIs was 82.6%; severe RSV-LRTIs reduction was 94.1%.	Pain, fatigue, muscle pain, headache, and joint stiffness or pain at the injection site; atrial fibrillation; Acute disseminated encephalomyelitis (ADEM; 2/2500); Guillain-Barre syndrome (1/2500).	Papi. et al. [40] Lee. et al. [41]
Abrysvo	Pregnant individuals	Approved by FDA	Against severe LRTIs was 81.8% (99.5% CI, 40.6 to 96.3); against RSV-associated LRIs was57.1% (99.5% CI, 14.7 to 79.8); against RSV-associated hospitalization within 180 days after birth was 56.8% (99.17% CI, 10.1 to 80.7).	Muscle pain and headache within 7 days after injection; pain in an arm followed by bilateral lower-extremity pain, premature labor, systemic lupus erythematosus, and eclampsia.	Kampmann. et al. [42] Syed. et al. [43] Fleming-Dutra. et al. [44] NCT04424316
**Recombinant vector vaccines**					
rBCG-N-hRSV	Newborns	Phase I	Safe, well-tolerated and no serious adverse events related to the vaccine. General solicited AEs: mild intensity (90.0%); moderate intensity (10.0%); headache (37.5%); fatigue (17.5%); diarrhea (15.0%); myalgia (12.5%). Two SAEs: grade 4 increase in CPK and one-day hospitalization, neither considered related to vaccine. Serum IgG-antibodies directed against Mycobacterium and the N-protein of RSV increased after vaccination. Cellular response increased starting at day 14 and 30 post-vaccination, respectively.	Pain was present in 23 individuals, erythema in 19, induration in 13, and lymphadenopathy in 2 (in both cases, lymphadenopathy was less than 2 cm, non-suppurative, and resolved without treatment). Erythema was detected more frequently with increasing doses of the study vaccine.	Rey-Jurado. et al. [45] Abarca. et al. [46] NCT03213405

**Table 2:** Evaluation of RSV Vaccines in Pediatric Research Utilizing Animal Models.

Animal Model	Age	Genotype	Vaccination Routes	Clinical Signs
Cotton rats	4–8 weeks	RSV A2	Intranasally	Hypertrophy of bronchial (mucous) epithelium, the presence of subepithelial inflammatory cells around bronchi (peribronchitis), bronchioles (peribronchiolitis) and blood vessels (perivasculitis) and in the alveoli (alveolitis)
	6 weeks	RSV A	Intramuscularly or intranasally	Infiltration of inflammatory cells in the peri-bronchus, thickening of the alveolar wall, and swelling of bronchial epithelial cells
	/	RSV A2	Intranasally	Incomplete protection against the heterotypic sub-group A RSV challenge.
AGM	4.2 years; 5.3–8.4 years	RSV A2	Intranasal and intratracheal	High level of replication of rA2; significant level of serum anti-RSV neutralizing antibody.
	4.2–8.4 years	RSV A2	Intranasal and intratracheal	Minimal to mild spots of lymphohistiocytic inflammation around terminal and respiratory bronchioles.
Foxp3-DTR/EGFP mice on a BALB/c background	6–8 weeks	RSV A2	Intramuscular injection; Subcutaneous	Mild lung pathology with some peribronchiolitis and nearly normal alveolar morphology
BALB/c mice	6–8 weeks	RSV A2	Subcutaneously in the scuff of the neck	Strong humoral and cellular immune responses.
	6–8 weeks	RSV A2	Subdermal injection	Infiltration of neutrophils in the airways; reduced neutrophil infiltration, viral loads and the number of activated memory CD8^+^ T cells after immunized; vaccine elicits a Th1/Th17 T cell repertoire that efficiently mediates virus clearance and prevents the inflammatory pathology in the lungs.
	10 weeks	RSV A2	Intranasal and intratracheal inoculation	Highly attenuated RSV strains replicate sporadically and at very low levels in mice.
	6–8 weeks	RSV A2	Intramuscularly	Robust Virus neutralizing antibodies titers (VNT) responses after RSV pre-exposed; vaccine induced robust cellular immune responses (IgG, CD8^+^ T cell, CD4^+^ T cell and RSV F-specific IFN-γ).

## Data Availability

No data were used for the research described in the article.

## References

[1] Falsey A.R., Williams K., Gymnopoulou E., Bart S., Ervin J., Bastian A.R., Menten J., De Paepe E., Vandenberghe S., Chan E.K.H. (2023). Efficacy and Safety of an Ad26.RSV.preF-RSV preF Protein Vaccine in Older Adults. N. Engl. J. Med..

[2] Li Y., Wang X., Blau D.M., Caballero M.T., Feikin D.R., Gill C.J., A Madhi S., Omer S.B., Simões E.A.F., Campbell H. (2022). Global, regional, and national disease burden estimates of acute lower respiratory infections due to respiratory syncytial virus in children younger than 5 years in 2019: A systematic analysis. Lancet.

[3] Hasso-Agopsowicz M., Hwang A., Hollm-Delgado M.G., Umbelino-Walker I., Karron R.A., Rao R., Asante K.P., Sheel M., Sparrow E., Giersing B. (2024). Identifying WHO global priority endemic pathogens for vaccine research and development (R&D) using multi-criteria decision analysis (MCDA): An objective of the Immunization Agenda 2030. eBioMedicine.

[4] World Health Organization (2024). WHO Study Lists Top Endemic Pathogens for Which New Vaccines are Urgently Needed. https://www.who.int/news/item/05-11-2024-who-study-lists-top-endemic-pathogens-for-which-new-vaccines-are-urgently-needed.

[5] Graham B.S., Sullivan N.J. (2018). Emerging viral diseases from a vaccinology perspective: Preparing for the next pandemic. Nat. Immunol..

[6] Kramer R., Duclos A., Lina B., Casalegno J.S., the VRS study group in Lyon (2018). Cost and burden of RSV related hospitalisation from 2012 to 2017 in the first year of life in Lyon, France. Vaccine.

[7] Demont C., Petrica N., Bardoulat I., Duret S., Watier L., Chosidow A., Lorrot M., Kieffer A., Lemaitre M. (2021). Economic and disease burden of RSV-associated hospitalizations in young children in France, from 2010 through 2018. BMC Infect. Dis..

[8] Openshaw P.J.M., Chiu C., Culley F.J., Johansson C. (2017). Protective and Harmful Immunity to RSV Infection. Annu. Rev. Immunol..

[9] Boyoglu-Barnum S., Chirkova T., Anderson L.J. (2019). Biology of Infection and Disease Pathogenesis to Guide RSV Vaccine Development. Front. Immunol..

[10] Asseri A.A. (2025). Respiratory Syncytial Virus: A Narrative Review of Updates and Recent Advances in Epidemiology, Pathogenesis, Diagnosis, Management and Prevention. J. Clin. Med..

[11] Guo L., Kenmoe S., Miyake F., Chung A., Zhang H., Bandeira T., Caballero M.T., Casalegno J.S., Fasce R., Giorgi C. (2025). Respiratory syncytial virus hospitalisation by chronological month of age and by birth month in infants. Nat. Commun..

[12] Eugenio B., Giovanni C.L., Claudio C., Heinrichs J.H., Manzoni P., Matteo Riccò M.R., Vassilouthis N. (2022). RSV disease in infants and young children: Can we see a brighter future?. Hum. Vaccines Immunother..

[13] Ong S. The Search for a Connection Between RSV and Asthma. https://www.nature.com/articles/d41586-023-02961-3.

[14] Verwey C., Madhi S.A. (2023). Review and Update of Active and Passive Immunization Against Respiratory Syncytial Virus. Biodrugs.

[15] Mao Z., Li X., Dacosta-Urbieta A., Billard M.N., Wildenbeest J., Korsten K., Martinon-Torres F., Heikkinen T., Cunningham S., Snape M.D. (2023). Economic burden and health-related quality-of-life among infants with respiratory syncytial virus infection: A multi-country prospective cohort study in Europe. Vaccine.

[16] Ajayi O.O., Ajufo A., Ekpa Q.L., Alabi P.O., Babalola F., Omar Z.T.O., Ekanem M., Ezuma-Ebong C., Ogunshola O.S., Akahara D.E. (2023). Evaluation of Bronchiolitis in the Pediatric Population in the United States of America and Canada: A Ten-Year Review. Cureus.

[17] Soni A., Kabra S.K., Lodha R. (2023). Respiratory Syncytial Virus Infection: An Update. Indian J. Pediatr..

[18] Michael B., Battles J.S.M. (2019). Respiratory syncytial virus entry and how to block it. Nat. Rev. Microbiol..

[19] Pickles R.J., DeVincenzo J.P. (2015). Respiratory syncytial virus (RSV) and its propensity for causing bronchiolitis. J. Pathol..

[20] Piralla A., Chen Z., Zaraket H. (2023). An update on respiratory syncytial virus. BMC Infect. Dis..

[21] Chen G., Ma X., Wu J., Yan Y., Qian W., Chen A., Yi C., Tian M. (2025). Host Immune Response to Respiratory Syncytial Virus Infection in Children. Influ. Other Respir. Viruses.

[22] Walsh E.E., Perez Marc G., Zareba A.M., Falsey A.R., Jiang Q., Patton M., Polack F.P., Llapur C., Doreski P.A., Ilangovan K. (2023). Efficacy and Safety of a Bivalent RSV Prefusion F Vaccine in Older Adults. N. Engl. J. Med..

[23] Allely D., Prasad V. (2023). Bivalent Prefusion F Vaccine in Pregnancy to Prevent RSV Illness in Infants. Reply. N. Engl. J. Med..

[24] Mazur N.I., Higgins D., Nunes M.C., Melero J.A., Langedijk A.C., Horsley N., Buchholz U.J., Openshaw P.J., McLellan J.S., Englund J.A. (2018). The respiratory syncytial virus vaccine landscape: Lessons from the graveyard and promising candidates. Lancet Infect. Dis..

[25] Belongia E.A., Simpson M.D., King J.P., Sundaram M.E., Kelley N.S., Osterholm M.T., McLean H.Q. (2016). Variable influenza vaccine effectiveness by subtype: A systematic review and meta-analysis of test-negative design studies. Lancet Infect. Dis..

[26] Dalziel S.R., Haskell L., O’Brien S., Borland M.L., Plint A.C., Babl F.E., Oakley E. (2022). Bronchiolitis. Lancet.

[27] Bulow S.M., Nir M., Levin E., Friis B., Thomsen L.L., Nielsen J.E., Holm J.C., Moller T., Bonde-Hansen M.E., Nielsen H.E. (1999). Prednisolone treatment of respiratory syncytial virus infection: A randomized controlled trial of 147 infants. Pediatrics.

[28] Rodriguez W.J., Gruber W.C., Groothuis J.R., Simoes E.A., Rosas A.J., Lepow M., Kramer A., Hemming V. (1997). Respiratory syncytial virus immune globulin treatment of RSV lower respiratory tract infection in previously healthy children. Pediatrics.

[29] Eichinger K.M., Kosanovich J.L., Lipp M., Empey K.M., Petrovsky N. (2021). Strategies for active and passive pediatric RSV immunization. Ther. Adv. Vaccines Immunother..

[30] Assad Z., Romain A.S., Aupiais C., Shum M., Schrimpf C., Lorrot M., Corvol H., Prevost B., Ferrandiz C., Giolito A. (2024). Nirsevimab and Hospitalization for RSV Bronchiolitis. N. Engl. J. Med..

[31] Caserta M.T., O’Leary S.T., Munoz F.M., Ralston S.L., Committee On Infectious Disease (2023). Palivizumab Prophylaxis in Infants and Young Children at Increased Risk of Hospitalization for Respiratory Syncytial Virus Infection. Pediatrics.

[32] Viguria N., Navascues A., Juanbeltz R., Echeverria A., Ezpeleta C., Castilla J. (2021). Effectiveness of palivizumab in preventing respiratory syncytial virus infection in high-risk children. Hum. Vaccines Immunother..

[33] Sevendal A.T.K., Hurley S., Bartlett A.W., Rawlinson W., Walker G.J. (2024). Systematic Review of the Efficacy and Safety of RSV-Specific Monoclonal Antibodies and Antivirals in Development. Rev. Med. Virol..

[34] Zar H.J., Simoes E.A.F., Madhi S.A., Ramilo O., Senders S.D., Shepard J.S., Laoprasopwattana K., Piedrahita J., Novoa J.M., Vargas S.L. (2025). Clesrovimab for Prevention of RSV Disease in Healthy Infants. N. Engl. J. Med..

[35] Syed Y.Y. (2025). Clesrovimab: First Approval. Drugs.

[36] Wilson E., Goswami J., Baqui A.H., Doreski P.A., Perez-Marc G., Zaman K., Monroy J., Duncan C.J.A., Ujiie M., Ramet M. (2023). Efficacy and Safety of an mRNA-Based RSV PreF Vaccine in Older Adults. N. Engl. J. Med..

[37] McFarland E.J., Karron R.A., Muresan P., Cunningham C.K., Libous J., Perlowski C., Thumar B., Gnanashanmugam D., Moye J., Schappell E. (2020). Live Respiratory Syncytial Virus Attenuated by M2-2 Deletion and Stabilized Temperature Sensitivity Mutation 1030s Is a Promising Vaccine Candidate in Children. J. Infect. Dis..

[38] Topalidou X., Kalergis A.M., Papazisis G. (2023). Respiratory Syncytial Virus Vaccines: A Review of the Candidates and the Approved Vaccines. Pathogens.

[39] Mazur N.I., Terstappen J., Baral R., Bardají A., Beutels P., Buchholz U.J., Cohen C., Crowe J.E., Cutland C.L., Eckert L. (2023). Respiratory syncytial virus prevention within reach: The vaccine and monoclonal antibody landscape. Lancet Infect. Dis..

[40] Papi A., Ison M.G., Langley J.M., Lee D.G., Leroux-Roels I., Martinon-Torres F., Schwarz T.F., van Zyl-Smit R.N., Campora L., Dezutter N. (2023). Respiratory Syncytial Virus Prefusion F Protein Vaccine in Older Adults. N. Engl. J. Med..

[41] Lee C.Y.F., Khan S.J., Vishal F., Alam S., Murtaza S.F. (2023). Respiratory Syncytial Virus Prevention: A New Era of Vaccines. Cureus.

[42] Kampmann B., Madhi S.A., Munjal I., Simoes E.A.F., Pahud B.A., Llapur C., Baker J., Perez Marc G., Radley D., Shittu E. (2023). Bivalent Prefusion F Vaccine in Pregnancy to Prevent RSV Illness in Infants. N. Engl. J. Med..

[43] Syed Y.Y. (2023). Respiratory Syncytial Virus Prefusion F Subunit Vaccine: First Approval of a Maternal Vaccine to Protect Infants. Paediatr. Drugs.

[44] Fleming-Dutra K.E., Jones J.M., Roper L.E., Prill M.M., Ortega-Sanchez I.R., Moulia D.L., Wallace M., Godfrey M., Broder K.R., Tepper N.K. (2023). Use of the Pfizer Respiratory Syncytial Virus Vaccine During Pregnancy for the Prevention of Respiratory Syncytial Virus-Associated Lower Respiratory Tract Disease in Infants: Recommendations of the Advisory Committee on Immunization Practices—United States, 2023. MMWR Morb. Mortal. Wkly. Rep..

[45] Rey-Jurado E., Bohmwald K., Correa H.G., Kalergis A.M. (2020). TCR Repertoire Characterization for T Cells Expanded in Response to hRSV Infection in Mice Immunized with a Recombinant BCG Vaccine. Viruses.

[46] Abarca K., Rey-Jurado E., Munoz-Durango N., Vazquez Y., Soto J.A., Galvez N.M.S., Valdes-Ferrada J., Iturriaga C., Urzua M., Borzutzky A. (2020). Safety and immunogenicity evaluation of recombinant BCG vaccine against respiratory syncytial virus in a randomized, double-blind, placebo-controlled phase I clinical trial. eClinicalMedicine.

[47] Gilman M.S., Castellanos C.A., Chen M., Ngwuta J.O., Goodwin E., Moin S.M., Mas V., Melero J.A., Wright P.F., Graham B.S. (2016). Rapid profiling of RSV antibody repertoires from the memory B cells of naturally infected adult donors. Sci. Immunol..

[48] McLellan J.S., Chen M., Leung S., Graepel K.W., Du X., Yang Y., Zhou T., Baxa U., Yasuda E., Beaumont T. (2013). Structure of RSV fusion glycoprotein trimer bound to a prefusion-specific neutralizing antibody. Science.

[49] Yu D., Zhang C., Qi Y., Liu Z., Yang D., Zhao N., Ke Z., Lu X., Li Y. (2025). RSV Vaccines: Targeting Prefusion F and G Proteins from Structural Design to Clinical Application. Vaccines.

[50] Hause A.M., Henke D.M., Avadhanula V., Shaw C.A., Tapia L.I., Piedra P.A. (2017). Sequence variability of the respiratory syncytial virus (RSV) fusion gene among contemporary and historical genotypes of RSV/A and RSV/B. PLoS ONE.

[51] Deng L., Cao H., Li G., Zhou K., Fu Z., Zhong J., Wang Z., Yang X. (2025). Progress on Respiratory Syncytial Virus Vaccine Development and Evaluation Methods. Vaccines.

[52] Killikelly A.M., Kanekiyo M., Graham B.S. (2016). Pre-fusion F is absent on the surface of formalin-inactivated respiratory syncytial virus. Sci. Rep..

[53] Sanchez-Martinez A., Moore T., Freitas T.S., Benzaken T.R., O’Hagan S., Millar E., Groves H.E., Drysdale S.B., Broadbent L. (2025). Recent advances in the prevention and treatment of respiratory syncytial virus disease. J. Gen. Virol..

[54] Anastassopoulou C., Medic S., Ferous S., Boufidou F., Tsakris A. (2025). Development, Current Status, and Remaining Challenges for Respiratory Syncytial Virus Vaccines. Vaccines.

[55] Brussow H. (2025). Respiratory syncytial virus: Health burden, disease prevention, and treatment-recent progress and lessons learned. Microlife.

[56] Swathi M. (2024). Arexvy: A Comprehensive Review of the Respiratory Syncytial Virus Vaccine for Revolutionary Protection. Viral Immunol..

[57] Alberto P., Michael G.I., Joanne M.L., Dong-Gun L., Leroux-Roels I., Federico Martinon-Torres T.F.S., van Zyl-Smit R.N., Campora L., Dezutter N., de Schrevel N. (2023). Safety and immunogenicity of a respiratory syncytial virus prefusion F (RSVPreF3) candidate vaccine in older adults: Phase I/II randomized clinical trial. N. Engl. J. Med..

[58] Stein R.T., Bont L.J., Zar H., Polack F.P., Park C., Claxton A., Borok G., Butylkova Y., Wegzyn C. (2017). Respiratory syncytial virus hospitalization and mortality: Systematic review and meta-analysis. Pediatr. Pulmonol..

[59] Goswami J., Cardona J.F., Caso J., Hsu D.C., Simorellis A.K., Wilson L., Dhar R., Wang X., Kapoor A., Collins A. (2025). Safety, Tolerability, and Immunogenicity of Revaccination with mRNA-1345, an mRNA Vaccine Against RSV, Administered 12 Months Following a Primary Dose in Adults Aged ≥50 Years. Clin. Infect. Dis..

[60] Anderson J., Do L.A.H., van Kasteren P.B., Licciardi P.V. (2024). The role of respiratory syncytial virus G protein in immune cell infection and pathogenesis. eBioMedicine.

[61] Tripp R.A., Power U.F., Openshaw P.J.M., Kauvar L.M. (2018). Respiratory Syncytial Virus: Targeting the G Protein Provides a New Approach for an Old Problem. J. Virol..

[62] Madi N., Sadeq M., Safar H.A., Al-Adwani A., Al-Turab M. (2024). Circulation of new lineages of RSV-A and RSV-B in Kuwait shows high diversity in the N- and O-linked glycosylation sites in the G protein between 2020 and 2022. Front. Cell. Infect. Microbiol..

[63] Anderson L.J., Jadhao S.J., Paden C.R., Tong S. (2021). Functional Features of the Respiratory Syncytial Virus G Protein. Viruses.

[64] Kopera E., Czajka H., Zapolnik P., Mazur A. (2023). New Insights on Respiratory Syncytial Virus Prevention. Vaccines.

[65] Abd-Eldaim M.M., Maarouf M., Potgieter L., Kania S.A. (2023). Amino acid variations of the immuno-dominant domain of respiratory syncytial virus attachment glycoprotein (G) affect the antibody responses In BALB/c mice. J. Virol. Methods.

[66] Sanz-Munoz I., Sanchez-de Prada L., Castrodeza-Sanz J., Eiros J.M. (2024). Microbiological and epidemiological features of respiratory syncytial virus. Rev. Esp. Quimioter..

[67] Lee Y., Klenow L., Coyle E.M., Grubbs G., Golding H., Khurana S. (2024). Monoclonal antibodies targeting sites in respiratory syncytial virus attachment G protein provide protection against RSV-A and RSV-B in mice. Nat. Commun..

[68] Sedeyn K., Schepens B., Saelens X. (2019). Respiratory syncytial virus nonstructural proteins 1 and 2: Exceptional disrupters of innate immune responses. PLoS Pathog..

[69] Gack M.U., Albrecht R.A., Urano T., Inn K.S., Huang I.C., Carnero E., Farzan M., Inoue S., Jung J.U., Garcia-Sastre A. (2009). Influenza A virus NS1 targets the ubiquitin ligase TRIM25 to evade recognition by the host viral RNA sensor RIG-I. Cell Host Microbe.

[70] Ban J., Lee N.R., Lee N.J., Lee J.K., Quan F.S., Inn K.S. (2018). Human Respiratory Syncytial Virus NS 1 Targets TRIM25 to Suppress RIG-I Ubiquitination and Subsequent RIG-I-Mediated Antiviral Signaling. Viruses.

[71] Goswami R., Majumdar T., Dhar J., Chattopadhyay S., Bandyopadhyay S.K., Verbovetskaya V., Sen G.C., Barik S. (2013). Viral degradasome hijacks mitochondria to suppress innate immunity. Cell Res..

[72] Bitko V., Shulyayeva O., Mazumder B., Musiyenko A., Ramaswamy M., Look D.C., Barik S. (2007). Nonstructural proteins of respiratory syncytial virus suppress premature apoptosis by an NF-kappaB-dependent, interferon-independent mechanism and facilitate virus growth. J. Virol..

[73] Thornhill E.M., Verhoeven D. (2020). Respiratory Syncytial Virus’s Non-structural Proteins: Masters of Interference. Front. Cell. Infect. Microbiol..

[74] Teng M.N. (2012). The non-structural proteins of RSV: Targeting interferon antagonists for vaccine development. Infect. Disord. Drug Targets.

[75] Churiso G., Husen G., Bulbula D., Abebe L. (2022). Immunity Cell Responses to RSV and the Role of Antiviral Inhibitors: A Systematic Review. Infect. Drug Resist..

[76] Lee Y.Z., Han J., Zhang Y.N., Ward G., Braz Gomes K., Auclair S., Stanfield R.L., He L., Wilson I.A., Zhu J. (2024). Rational design of uncleaved prefusion-closed trimer vaccines for human respiratory syncytial virus and metapneumovirus. Nat. Commun..

[77] Diaz F.E., Guerra-Maupome M., McDonald P.O., Rivera-Perez D., Kalergis A.M., McGill J.L. (2021). A Recombinant BCG Vaccine Is Safe and Immunogenic in Neonatal Calves and Reduces the Clinical Disease Caused by the Respiratory Syncytial Virus. Front. Immunol..

[78] Georgakopoulou V.E., Pitiriga V.C. (2025). Immunomodulation in Respiratory Syncytial Virus Infection: Mechanisms, Therapeutic Targets, and Clinical Implications. Microorganisms.

[79] Acosta P.L., Caballero M.T., Polack F.P. (2015). Brief History and Characterization of Enhanced Respiratory Syncytial Virus Disease. Clin. Vaccine Immunol..

[80] Kim H.W., Canchola J.G., Brandt C.D., Pyles G., Chanock R.M., Jensen K., Parrott R.H. (1969). Respiratory syncytial virus disease in infants despite prior administration of antigenic inactivated vaccine. Am. J. Epidemiol..

[81] Boelen A., Andeweg A., Kwakkel J., Lokhorst W., Bestebroer T., Dormans J., Kimman T. (2000). Both immunisation with a formalin-inactivated respiratory syncytial virus (RSV) vaccine and a mock antigen vaccine induce severe lung pathology and a Th2 cytokine profile in RSV-challenged mice. Vaccine.

[82] Frenkel L.D., Gaur S., Bellanti J.A. (2023). The third pandemic: The respiratory syncytial virus landscape and specific considerations for the allergist/immunologist. Allergy Asthma Proc..

[83] Johnson T.R., Rao S., Seder R.A., Chen M., Graham B.S. (2009). TLR9 agonist, but not TLR7/8, functions as an adjuvant to diminish FI-RSV vaccine-enhanced disease, while either agonist used as therapy during primary RSV infection increases disease severity. Vaccine.

[84] Knudson C.J., Hartwig S.M., Meyerholz D.K., Varga S.M. (2015). RSV vaccine-enhanced disease is orchestrated by the combined actions of distinct CD4 T cell subsets. PLoS Pathog..

[85] Russell C.D., Unger S.A., Walton M., Schwarze J. (2017). The Human Immune Response to Respiratory Syncytial Virus Infection. Clin. Microbiol. Rev..

[86] Reed J.L., Welliver T.P., Sims G.P., McKinney L., Velozo L., Avendano L., Hintz K., Luma J., Coyle A.J., Welliver R.C. (2009). Innate immune signals modulate antiviral and polyreactive antibody responses during severe respiratory syncytial virus infection. J. Infect. Dis..

[87] Chiu C., Ellebedy A.H., Wrammert J., Ahmed R. (2015). B cell responses to influenza infection and vaccination. Curr. Top. Microbiol. Immunol..

[88] Habibi M.S., Jozwik A., Makris S., Dunning J., Paras A., DeVincenzo J.P., de Haan C.A., Wrammert J., Openshaw P.J., Chiu C. (2015). Impaired Antibody-mediated Protection and Defective IgA B-Cell Memory in Experimental Infection of Adults with Respiratory Syncytial Virus. Am. J. Respir. Crit. Care Med..

[89] An L.L., Whitton J.L. (1997). A multivalent minigene vaccine, containing B-cell, cytotoxic T-lymphocyte, and Th epitopes from several microbes, induces appropriate responses in vivo and confers protection against more than one pathogen. J. Virol..

[90] Kruijsen D., Bakkers M.J., van Uden N.O., Viveen M.C., van der Sluis T.C., Kimpen J.L., Leusen J.H., Coenjaerts F.E., van Bleek G.M. (2010). Serum antibodies critically affect virus-specific CD4+/CD8+ T cell balance during respiratory syncytial virus infections. J. Immunol..

[91] Fan C.F., Zeng R.H., Sun C.Y., Mei X.G., Wang Y.F., Liu Y. (2005). Fusion of DsbA to the N-terminus of CTL chimeric epitope, F/M2:81-95, of respiratory syncytial virus prolongs protein- and virus-specific CTL responses in Balb/c mice. Vaccine.

[92] Didierlaurent A.M., Laupeze B., Di Pasquale A., Hergli N., Collignon C., Garcon N. (2017). Adjuvant system AS01: Helping to overcome the challenges of modern vaccines. Expert Rev. Vaccines.

[93] Roman F., Burny W., Ceregido M.A., Laupeze B., Temmerman S.T., Warter L., Coccia M. (2024). Adjuvant system AS01: From mode of action to effective vaccines. Expert Rev. Vaccines.

[94] Stertman L., Palm A.E., Zarnegar B., Carow B., Lunderius Andersson C., Magnusson S.E., Carnrot C., Shinde V., Smith G., Glenn G. (2023). The Matrix-M adjuvant: A critical component of vaccines for the 21(st) century. Hum. Vaccines Immunother..

[95] Strannegård Ö., Cello J., Bjarnason R., Sigurbergsson F., Sigurs N. (1997). Association between pronounced IgA response in RSV bronchiolitis and development of allergic sensitization. Pediatr. Allergy Immunol..

[96] Gote V., Bolla P.K., Kommineni N., Butreddy A., Nukala P.K., Palakurthi S.S., Khan W. (2023). A Comprehensive Review of mRNA Vaccines. Int. J. Mol. Sci..

[97] Terstappen J., Hak S.F., Bhan A., Bogaert D., Bont L.J., Buchholz U.J., Clark A.D., Cohen C., Dagan R., Feikin D.R. (2024). The respiratory syncytial virus vaccine and monoclonal antibody landscape: The road to global access. Lancet Infect. Dis..

[98] Aldosari B.N., Alfagih I.M., Almurshedi A.S. (2021). Lipid Nanoparticles as Delivery Systems for RNA-Based Vaccines. Pharmaceutics.

[99] Li S., Zheng L., Zhong J., Gao X. (2025). Advancing mRNA vaccines for infectious diseases: Key components, innovations, and clinical progress. Essays Biochem..

[100] van Drunen Littel-van den Hurk S., Mapletoft J.W., Arsic N., Kovacs-Nolan J. (2007). Immunopathology of RSV infection: Prospects for developing vaccines without this complication. Rev. Med. Virol..

[101] Odio C.D., Katzelnick L.C. (2022). ‘Mix and Match’ vaccination: Is dengue next?. Vaccine.

[102] Piano Mortari E., Ferrucci F., Zografaki I., Carsetti R., Pacelli L. (2025). T and B cell responses in different immunization scenarios for COVID-19: A narrative review. Front. Immunol..

[103] Lakerveld A.J., Gelderloos A.T., Schepp R.M., de Haan C.A.M., van Binnendijk R.S., Rots N.Y., van Beek J., van Els C., van Kasteren P.B. (2023). Difference in respiratory syncytial virus-specific Fc-mediated antibody effector functions between children and adults. Clin. Exp. Immunol..

[104] Pollard A.J., Bijker E.M. (2021). A guide to vaccinology: From basic principles to new developments. Nat. Rev. Immunol..

[105] Keam S.J. (2023). Nirsevimab: First Approval. Drugs.

[106] Sealy R.E., Surman S.L., Hurwitz J.L. (2017). CD4(+) T cells support establishment of RSV-specific IgG and IgA antibody secreting cells in the upper and lower murine respiratory tract following RSV infection. Vaccine.

[107] Bai H., Yu X., Gao Y., Li Q., Wen B., Hu R. (2025). Highly Effective mRNA-LNP Vaccine Against Respiratory Syncytial Virus (RSV) in Multiple Models. Vaccines.

[108] Etti M., Calvert A., Galiza E., Lim S., Khalil A., Le Doare K., Heath P.T. (2022). Maternal vaccination: A review of current evidence and recommendations. Am. J. Obstet. Gynecol..

[109] Patel D., Chawla J., Blavo C. (2024). Use of the Abrysvo Vaccine in Pregnancy to Prevent Respiratory Syncytial Virus in Infants: A Review. Cureus.

[110] Abbasi H.Q., Oduoye M.O. (2023). Revitalizing hope for older adults: The use of the novel Arexvy for immunization against respiratory syncytial virus. Health Sci. Rep..

[111] Hermida N., Ferguson M., Leroux-Roels I., Pagnussat S., Yaplee D., Hua N., van den Steen P., Anspach B., Dieussaert I., Kim J.H. (2024). Safety and Immunogenicity of Respiratory Syncytial Virus Prefusion Maternal Vaccine Coadministered With Diphtheria-Tetanus-Pertussis Vaccine: A Phase 2 Study. J. Infect. Dis..

[112] Simoes E.A.F., Bont L., Manzoni P., Fauroux B., Paes B., Figueras-Aloy J., Checchia P.A., Carbonell-Estrany X. (2018). Past, Present and Future Approaches to the Prevention and Treatment of Respiratory Syncytial Virus Infection in Children. Infect. Dis. Ther..

[113] Resch B. (2017). Product review on the monoclonal antibody palivizumab for prevention of respiratory syncytial virus infection. Hum. Vaccines Immunother..

[114] Domachowske J.B. (2024). New and Emerging Passive Immunization Strategies for the Prevention of RSV Infection During Infancy. J. Pediatric. Infect. Dis. Soc..

[115] Mapindra M.P., Castillo-Hernandez T., Clark H., Madsen J. (2025). Surfactant Protein-A and its immunomodulatory roles in infant respiratory syncytial virus infection: A potential for therapeutic intervention?. Am. J. Physiol. Lung Cell. Mol. Physiol..

[116] Harder O.E., Niewiesk S. (2022). Respiratory Syncytial Virus Infection Modeled in Aging Cotton Rats (*Sigmodon hispidus*) and Mice (*Mus musculus*). Adv. Virol..

[117] Bem R.A., Domachowske J.B., Rosenberg H.F. (2011). Animal models of human respiratory syncytial virus disease. Am. J. Physiol. Lung Cell. Mol. Physiol..

[118] Collins P.L., Murphy B.R. (2005). New generation live vaccines against human respiratory syncytial virus designed by reverse genetics. Proc. Am. Thorac. Soc..

[119] Karron R.A., Buchholz U.J., Collins P.L. (2013). Live-attenuated respiratory syncytial virus vaccines. Curr. Top. Microbiol. Immunol..

[120] Taylor G. (2017). Animal models of respiratory syncytial virus infection. Vaccine.

[121] Brock L.G., Liu X., Liang B., Lingemann M., Liu X., Herbert R., Hackenberg A.D., Buchholz U.J., Collins P.L., Munir S. (2018). Murine Pneumonia Virus Expressing the Fusion Glycoprotein of Human Respiratory Syncytial Virus from an Added Gene Is Highly Attenuated and Immunogenic in Rhesus Macaques. J. Virol..

[122] Jones B.G., Sealy R.E., Rudraraju R., Traina-Dorge V.L., Finneyfrock B., Cook A., Takimoto T., Portner A., Hurwitz J.L. (2012). Sendai virus-based RSV vaccine protects African green monkeys from RSV infection. Vaccine.

[123] Saeland E., van der Fits L., Bolder R., Heemskerk-van der Meer M., Drijver J., van Polanen Y., Vaneman C., Tettero L., Serroyen J., Schuitemaker H. (2022). Immunogenicity and protective efficacy of adenoviral and subunit RSV vaccines based on stabilized prefusion F protein in pre-clinical models. Vaccine.

[124] Chen Y., Green S.R., Almazan F., Quehenberger O. (2006). The amino terminus and the third extracellular loop of CX3CR1 contain determinants critical for distinct receptor functions. Mol. Pharmacol..

[125] Anderson C.S., Chu C.Y., Wang Q., Mereness J.A., Ren Y., Donlon K., Bhattacharya S., Misra R.S., Walsh E.E., Pryhuber G.S. (2020). CX3CR1 as a respiratory syncytial virus receptor in pediatric human lung. Pediatr. Res..

[126] Blanco J.C.G., Cullen L.M., Kamali A., Sylla F.Y.D., Boukhvalova M.S., Morrison T.G. (2021). Evolution of protection after maternal immunization for respiratory syncytial virus in cotton rats. PLoS Pathog..

[127] Lin M., Yin Y., Zhao X., Wang C., Zhu X., Zhan L., Chen L., Wang S., Lin X., Zhang J. (2025). A truncated pre-F protein mRNA vaccine elicits an enhanced immune response and protection against respiratory syncytial virus. Nat. Commun..

[128] Boukhvalova M., Blanco J.C., Falsey A.R., Mond J. (2016). Treatment with novel RSV Ig RI-002 controls viral replication and reduces pulmonary damage in immunocompromised *Sigmodon hispidus*. Bone Marrow Transplant..

[129] Xiong R., Fu R., Wu Y., Wu X., Cao Y., Qu Z., Yang Y., Liu S., Huo G., Wang S. (2022). Long-Term Infection and Pathogenesis in a Novel Mouse Model of Human Respiratory Syncytial Virus. Viruses.

[130] Anderson J.J., Norden J., Saunders D., Toms G.L., Scott R. (1990). Analysis of the local and systemic immune responses induced in BALB/c mice by experimental respiratory syncytial virus infection. J. Gen. Virol..

[131] Openshaw P.J. (2013). The mouse model of respiratory syncytial virus disease. Curr. Top. Microbiol. Immunol..

[132] Taylor G., Stott E.J., Bew M., Fernie B.F., Cote P.J., Collins A.P., Hughes M., Jebbett J. (1984). Monoclonal antibodies protect against respiratory syncytial virus infection in mice. Immunology.

[133] Kim M.J., Chu K.B., Lee S.H., Mao J., Eom G.D., Yoon K.W., Moon E.K., Quan F.S. (2024). Assessing the protection elicited by virus-like particles expressing the RSV pre-fusion F and tandem repeated G proteins against RSV rA2 line19F infection in mice. Respir. Res..

[134] Xu Y., Sun F., Bai Z., Bian C., Wang X., Zhao Z., Yang P. (2024). Cold-adapted influenza-vectored RSV vaccine protects BALB/c mice and cotton rats from RSV challenge. J. Med. Virol..

[135] Zhan L., Zhao M., Yi H., Zhang W., Cao J., Sun Y., Zhang L., Si J., Xia N., Zheng Z. (2016). Comparison of Respiratory Syncytial Virus Infection on Different Week-ages BALB/c Mice. Chin. J. Virol..

[136] Graham B.S., Perkins M.D., Wright P.F., Karzon D.T. (1988). Primary respiratory syncytial virus infection in mice. J. Med. Virol..

[137] DeVincenzo J.P., Wilkinson T., Vaishnaw A., Cehelsky J., Meyers R., Nochur S., Harrison L., Meeking P., Mann A., Moane E. (2010). Viral load drives disease in humans experimentally infected with respiratory syncytial virus. Am. J. Respir. Crit. Care Med..

[138] Johnson J.E., Gonzales R.A., Olson S.J., Wright P.F., Graham B.S. (2007). The histopathology of fatal untreated human respiratory syncytial virus infection. Mod. Pathol..

[139] Sharma A., Wu W., Sung B., Huang J., Tsao T., Li X., Gomi R., Tsuji M., Worgall S. (2016). Respiratory Syncytial Virus (RSV) Pulmonary Infection in Humanized Mice Induces Human Anti-RSV Immune Responses and Pathology. J. Virol..

[140] Harker J.A., Yamaguchi Y., Culley F.J., Tregoning J.S., Openshaw P.J. (2014). Delayed sequelae of neonatal respiratory syncytial virus infection are dependent on cells of the innate immune system. J. Virol..

[141] Griffiths C.D., Bilawchuk L.M., McDonough J.E., Jamieson K.C., Elawar F., Cen Y., Duan W., Lin C., Song H., Casanova J.L. (2020). IGF1R is an entry receptor for respiratory syncytial virus. Nature.

[142] You D., Becnel D., Wang K., Ripple M., Daly M., Cormier S.A. (2006). Exposure of neonates to respiratory syncytial virus is critical in determining subsequent airway response in adults. Respir. Res..

[143] You D., Saravia J., Siefker D., Shrestha B., Cormier S.A. (2016). Crawling with Virus: Translational Insights from a Neonatal Mouse Model on the Pathogenesis of Respiratory Syncytial Virus in Infants. J. Virol..

[144] Cormier S.A., Shrestha B., Saravia J., Lee G.I., Shen L., DeVincenzo J.P., Kim Y.I., You D. (2014). Limited type I interferons and plasmacytoid dendritic cells during neonatal respiratory syncytial virus infection permit immunopathogenesis upon reinfection. J. Virol..

[145] Mandviwala A.S., Huckriede A.L.W., Arankalle V.A., Patil H.P. (2024). Mucosal delivery of a prefusogenic-F, glycoprotein, and matrix proteins-based virus-like particle respiratory syncytial virus vaccine induces protective immunity as evidenced by challenge studies in mice. Virology.

[146] Bian L., Zheng Y., Guo X., Li D., Zhou J., Jing L., Chen Y., Lu J., Zhang K., Jiang C. (2022). Intramuscular Inoculation of AS02-Adjuvanted Respiratory Syncytial Virus (RSV) F Subunit Vaccine Shows Better Efficiency and Safety Than Subcutaneous Inoculation in BALB/c Mice. Front. Immunol..

[147] Rivas-Fuentes S., Salgado-Aguayo A., Santos-Mendoza T., Sevilla-Reyes E. (2024). The Role of the CX3CR1-CX3CL1 Axis in Respiratory Syncytial Virus Infection and the Triggered Immune Response. Int. J. Mol. Sci..

[148] Zhao C., Taliento A.E., Belkin E.M., Fearns R., Lerou P.H., Ai X., Bai Y. (2025). Infant RSV infection desensitizes beta2-adrenergic receptor via CXCL11-CXCR7 signaling in airway smooth muscle. bioRxiv.

[149] Deo V.K., Tsuji Y., Yasuda T., Kato T., Sakamoto N., Suzuki H., Park E.Y. (2011). Expression of an RSV-gag virus-like particle in insect cell lines and silkworm larvae. J. Virol. Methods.

[150] Mejias A., Rodríguez-Fernández R., Oliva S., Peeples M.E., Ramilo O. (2021). The Journey to an RSV Vaccine. Ann. Allergy Asthma Immunol..

[151] Jordan E., Jenkins V., Silbernagl G., Chavez M.P.V., Schmidt D., Schnorfeil F., Schultz S., Chen L., Salgado F., Jacquet J.M. (2024). A multivalent RSV vaccine based on the modified vaccinia Ankara vector shows moderate protection against disease caused by RSV in older adults in a phase 3 clinical study. Vaccine.

[152] Samy N., Reichhardt D., Schmidt D., Chen L.M., Silbernagl G., Vidojkovic S., Meyer T.P., Jordan E., Adams T., Weidenthaler H. (2020). Safety and immunogenicity of novel modified vaccinia Ankara-vectored RSV vaccine: A randomized phase I clinical trial. Vaccine.

[153] A Phase III Double-blind Study to Assess Safety and Efficacy of an RSV Maternal Unadjuvanted Vaccine, in Pregnant Women and Infants Born to Vaccinated Mothers (GRACE). https://clinicaltrials.gov/study/NCT04605159.

[154] FDA Cases of Severe RSV in Two Moderna Vaccine Candidates’ Trials Raise Questions About Future. https://endpts.com/cases-of-severe-rsv-in-two-moderna-vaccine-candidates-trials-raise-questions-about-future/.

[155] FDA (2024). Preliminary Analysis of Guillain-Barré Syndrome (GBS) Following RSV Vaccination Among Adults 65 Years and Older. https://www.cdc.gov/acip/downloads/slides-2024-02-28-29/06-RSV-Adults-Lloyd--508.pdf.

